# Efficacy and safety of acupoint catgut embedding in treating postoperative pain of mixed hemorrhoids: A randomized controlled trial protocol

**DOI:** 10.1097/MD.0000000000025948

**Published:** 2021-05-14

**Authors:** Xiaorui Pei, Shijun Song, Haotian Li, Debao Lu

**Affiliations:** a Department of General Surgery, Tianjin TEDA Hospital; b Department of Hepatobiliary and Pancreatic Surgery, Tianjin Nankai Hospital, Tianjin, China.

**Keywords:** acupoint catgut embedding, mixed hemorrhoids, postoperative pain, protocol, randomized controlled trial

## Abstract

**Background:**

Pain is a common complication after mixed hemorrhoids, which seriously affects the recovery of patients and prolongs the length of hospital stay. Acupoint catgut embedding has advantages in improving a variety of acute and chronic pain diseases, but there is still a lack of rigorous randomized controlled studies to verify its efficacy and safety in the treatment of postoperative pain of mixed hemorrhoids. Therefore, the purpose of this randomized controlled trial is to evaluate the clinical efficacy of acupoint catgut embedding in the treatment of postoperative pain of mixed hemorrhoids.

**Methods:**

This is a prospective randomized controlled trial to study the efficacy and safety of acupoint catgut embedding in the treatment of postoperative pain of mixed hemorrhoids. Approved by the clinical research ethics committee of our hospital, the patients were randomly divided into observation group and control group according to 1:1. The observation group received acupoint catgut embedding before the operation, while the control group received no special treatment. The efficacy and safety indexes were concerned after the operation, and the observation indexes included: resting state and visual analogue scale (VAS) score during defecation, postoperative hospitalization time, total amount of analgesic use, adverse reactions, etc. Finally, we carried on the data statistical analysis through the SPSS version 19.0.

**Discussion:**

This study will evaluate the efficacy and safety of acupoint catgut embedding in the treatment of postoperative pain of mixed hemorrhoids, and the results of this study will provide a new idea for the selection of postoperative analgesia for mixed hemorrhoids resection.

**Trial registration:**

OSF Registration number: DOI 10.17605/OSF.IO/T2ZGY.

## 1 Introduction

Hemorrhoids is one of the most common diseases in anorectal diseases, which can occur at any age.^[1]^ With the acceleration of social aging, the incidence of hemorrhoids increases with age, and women are higher than men's,^[2]^ affecting millions of people in the world, is a major medical and social economic problem.^[3]^ The treatment of hemorrhoids depends on the symptoms and stages of the disease. Hemorrhoidectomy is considered to be an effective method for the treatment of III-IV degree symptomatic hemorrhoids.^[4]^ Open hemorrhoidectomy is still the most common surgery for hemorrhoids, despite the emergence of many surgical methods.^[5]^ Pain is a common complication after hemorrhoidectomy, with moderate and severe pain accounting for 65% of the total number of patients,^[6]^ affecting patients’ recovery and prolonging hospital stay.

Opioid painkillers and non-steroidal anti-inflammatory drugs are commonly used to relieve pain, but their action time is short and have side effects.^[7,8]^ Some other methods have been also used to reduce postoperative pain, such as COX-2-specific inhibitors, botulinum toxin, and calcium channel blockers, etc.,^[9,10]^ but there are side effects or there is no conclusive evidence to prove their efficacy.^[5,11]^ Therefore, it is necessary to continue to explore safe and effective solutions to the problem of postoperative pain in patients with hemorrhoidectomy.

Acupoint catgut embedding is a traditional external therapy in China, which is an extension and development of acupuncture and moxibustion therapy. Under the guidance of meridian theory, catgut or absorbable surgical thread is implanted into the corresponding acupoints, and the decomposition and absorption process produces a lasting, slow and stable acupuncture effect at the acupoints. At present, it has been widely used in the treatment of acute and chronic pain such as migraine, sciatica and low back pain,^[12–14]^ with the characteristics of certain curative effect, low cost and low adverse reactions. Now there is evidence that electroacupuncture combined with acupoint catgut embedding can effectively improve postoperative pain of mixed hemorrhoids and reduce the use of analgesics.^[15]^ However, there is a lack of a single randomized controlled study on acupoint catgut embedding in the treatment of postoperative pain of mixed hemorrhoids. Therefore, this randomized controlled trial is intended to evaluate the efficacy and safety of acupoint catgut embedding in the treatment of postoperative pain of mixed hemorrhoids.

## 2 Materials and methods

### 2.1 Study design

**Figure 1:**
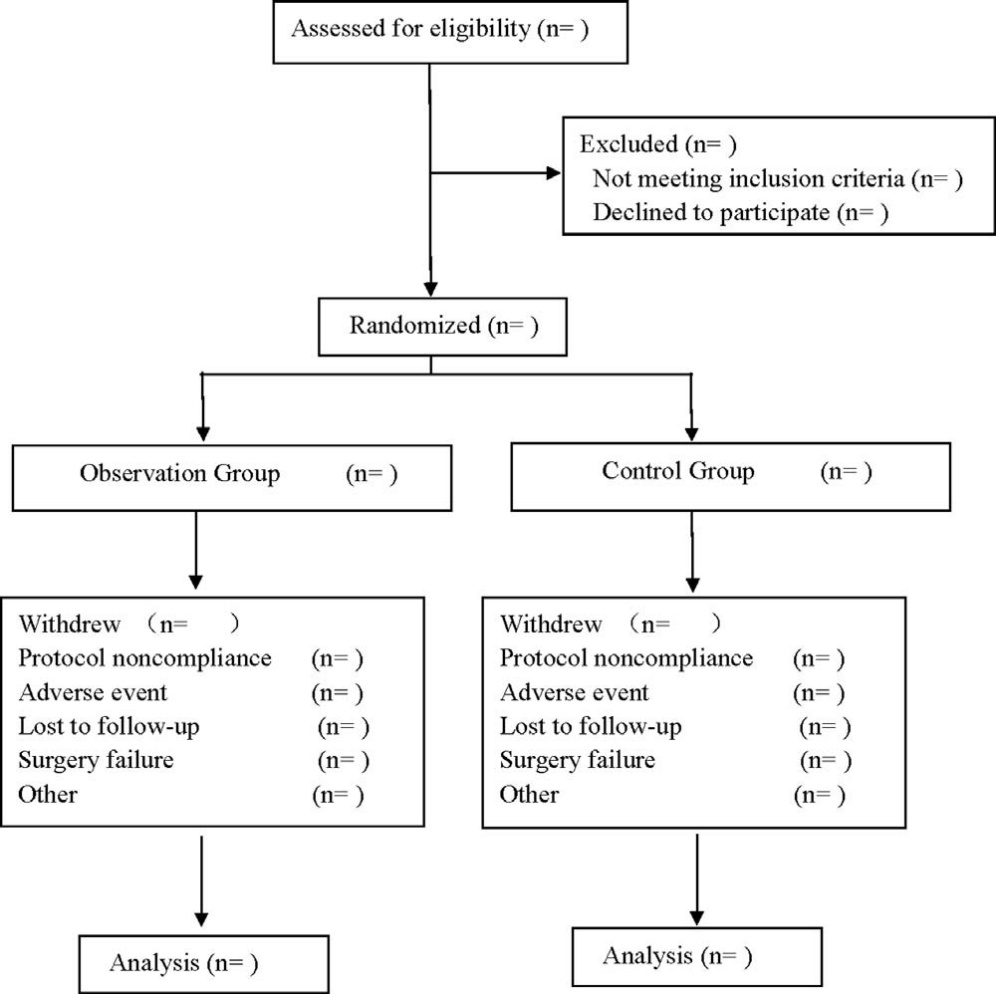
Flow diagram.

This is a prospective, single-blind, randomized controlled trial to investigate the efficacy and safety of acupoint catgut embedding in the treatment of postoperative pain of mixed hemorrhoids. This trial will follow the comprehensive trial reporting criteria,^[16]^ and the flow chart is shown in Figure 1.

### 2.2 Ethics and registration

This study protocol was in accordance with the Helsinki Declaration and approved by the Clinical Research Ethics Committee of our hospital. This experiment has been registered at open science framework (registration number: DOI 10.17605/OSF.IO/T2ZGY). Prior to randomization, all patients were required to sign a written informed consent form. Participants could withdraw from the study at any time for any reason, and patients’ consent would be required for their data to be used after the withdrawal.

### 2.3 Patients

Inclusion criteria: ① In accordance with the diagnosis of mixed hemorrhoids, and belong to stage III and stage VI (diagnostic criteria referring to *Clinical Guidelines for Diagnosis and Treatment of Hemorrhoids (2006)*^[17]^ formulated by Colorectal and Anorectal Surgery Group of Chinese Medical Association, etc.); ②Aging 18 to 65 years old; ③With no previous hemorrhoid surgery history, and the shape and function of the anus was completely normal; ④Patients with voluntary test, signed informed consent and having a good compliance.

Exclusion criteria: ① Patients with previous history of anal surgery; ②Patients with other rectal and anal diseases, such as perianal abscess, rectal polyps, rectal tumors, etc; ③Patients with sacrococcygeal injury, and local skin rupture was not suitable for embedding ④Pregnant women, lactating women and allergic people; ⑤Patients who had used analgesics within 24 hours before surgery; ⑥Patients with allergy or contraindications to the medication used in this study.

### 2.4 Sample size

The sample size was calculated based on the mean and standard deviation of the VAS scores in the resting state of patients 12 hours after surgery. According to the results of the pilot study, the observation group was 3.58 ± 1.54, the control group was 4.35 ± 1.38. Set α=0.025, one-tailed test, β=0.20. According to the calculation of PASS15.0 software, 58 participants were required for each group, and the estimated withdrawal rate was 10%. 65 participants would be included in each group.

### 2.5 Randomization and blinding

Patients included in the study were randomly divided into an observation or treatment group according to the 1:1 ratio. An independent statistician was used to randomly group the patients, and the random sequences were generated using SAS V.9.4 software, which would be enclosed in sealed, opaque envelopes with numbered numbers. The patients randomly selected the envelopes and were grouped according to the sequences within the envelopes. The allocation of patients, research assistants, statistical analysts, and nurses to the study was unknowable throughout the study. Due to the limitations of the type of intervention, the surgeon was aware of the allocation.

### 2.6 Study design

All patients would undergo the Milligan-Morgan technique of open hemorrhoidectomy by the same surgical team under general anesthesia. In order to ensure the consistency of surgical techniques, all patients were operated by a same surgeon, and all patients used a unified anesthetic plan, and the resection process was the same in both groups.

After the anesthesia took effect before the operation, the observation group performed acupoint catgut embedding. After disinfecting Chengshan (BL57) point and Changqiang (DU1) point area, we used lumbar puncture needle cannula 26 milli-needle, put 1 cm of catgut in the front end, inserted the needle core from the tail end, punctured the acupoint to get qi, slowly pushed into the needle core, and withdrew from the needle tube, and kept the catgut in the acupoint. The mixed hemorrhoidectomy was carried out after embedding the thread.

In the control group, mixed hemorrhoidectomy was performed directly after anesthesia took effect.

Both groups of patients were treated with the same postoperative nursing plan. When the pain was unbearable, they were given Nimesulide dispersible tablet 0.1 g orally (Wuhan Changming Pharmaceutical Co., Ltd., China, National Drug Approval No. H20010730), and the usage was recorded.

### 2.7 Evaluation criteria and efficacy evaluation

1. Primary outcome measures: the visual analogue scale^[18]^ (VAS), which is commonly used to measure pain intensity, would be used to evaluate changes in patient pain.^[19]^ Patients would be asked to mark a point between 0 and 100, where 100 means the most pain (the far right) and 0 means no pain (the far left). VAS scores at resting state were recorded at 4 hours, 12 hours, 24 hours, and 48 hours after surgery. VAS scores during defecation were recorded on postoperative day 1, day 2, day 3, and day 7.
2. Secondary outcomes: ① total amount (tablets) of Nimesulide dispersible tablets taken orally by the patient within 7 days after surgery; ②patients’ stay in hospital after operation (defined as the day from the start of operation to discharge); ③adverse reactions: patients in both groups experienced discomfort (such as skin itching, redness, nausea, dizziness, etc.) during treatment.

### 2.8 Data collection and management

All research data would be collected and collated by 1 to 2 research assistants and recorded in pre-designed tables. The study materials would be kept in a separate storage room to protect confidentiality before, during and after the study. Access to the database was limited to researchers in the research group.

### 2.9 Statistical analysis

SPSS19.0 statistical software was used to process the data, the measurement data were expressed as mean ± standard deviation ( ± S), the *t* test was used, the counting data was expressed as rate (%), and the *χ*^2^ test was applied. When *P* < .05, the difference was statistically significant.

## 3 Discussion

The inner wall of the anal canal is one of the most innervated tissues in the digestive tract, so the occurrence of pain after hemorrhoidectomy is a predictable outcome.^[20]^ The degree of pain is affected by many factors, including surgical methods, defecation, spasm of anal sphincter, perianal inflammation, etc.^[5,20]^ The pain may persist throughout the whole treatment, and the wound pain during dressing change increases the patient's fear. Some patients even dare not defecate because of fear of pain, causing constipation, causing a lot of trouble.

Acupoint catgut embedding is a compound treatment method integrating multiple effects, such as acupoint sealing effect, acupuncture effect, pricking blood effect and embedding needle effect, with distinct characteristics of traditional Chinese medicine.^[21]^ Modern studies have confirmed that it has the following functions:^[22–24]^① Adjusting immune function: the absorbable line implanted into the acupoints by thread embedment therapy is a foreign protein for the body, which can stimulate the body to produce allergic reactions, produce lymphatic factors, improve the phagocytic function of macrophages, so as to enhance the immune function of the body; ② Enhance the ability of stress: the stimulation of the absorbable line embedded in the acupoints on the body can improve the stress ability of the body, promote local vasodilation and increase neovascularization beds, increase blood flow, improve local lymphatic and blood circulation, accelerate the metabolism of tissue, thus reducing exudative adhesions and accelerating absorption of inflammation; ③ Promote the generation of neuropeptide substances: acupoint catgut embedding can stimulate the central nervous system to produce nerve impulse, promote the secretion and synthesis of endogenous opioid substances such as endorphins, improve the pain threshold, and block the feedback loop of pain signals by inhibiting the release of inflammatory factors such as nitric oxide, so as to achieve the effect of pain relief. At present, acupoint catgut embedding has been applied to a variety of pain diseases, and its efficacy and safety have been confirmed.^[24]^ We intend to explore the efficacy of acupoint catgut embedding in the treatment of postoperative pain of mixed hemorrhoids through this randomized controlled study, and the results of this study will provide a new idea for the selection of postoperative analgesia after mixed hemorrhoidectomy.

This study also has some limitations: due to the short observation period in this study, we cannot understand the impact of long-term efficacy, so we may increase the follow-up time if necessary; due to the particularity of the intervention measures, this study could not achieve double blindness, which may have some impact on the results.

## Author contributions

**Conceptualization:** Xiaorui Pei, Haotian Li.

**Data curation:** Xiaorui Pei, Shijun Song.

**Formal analysis:** Shijun Song, Haotian Li.

**Funding acquisition:** Debao Lu.

**Software:** Haotian Li, Debao Lu.

**Supervision:** Haotian Li.

**Writing – original draft:** Xiaorui Pei, Shijun Song.

**Writing – review & editing:** Xiaorui Pei, Shijun Song.
